# Design, expression and functional characterization of a thermostable xylanase from *Trichoderma reesei*

**DOI:** 10.1371/journal.pone.0210548

**Published:** 2019-01-16

**Authors:** Jun He, Feng Tang, Daiwen Chen, Bing Yu, Yuheng Luo, Ping Zheng, Xiangbing Mao, Jie Yu, Feng Yu

**Affiliations:** Institute of Animal Nutrition, Sichuan Agricultural University, Chengdu, Sichuan, P. R. China; Weizmann Institute of Science, ISRAEL

## Abstract

Xylanases isolated from microorganisms such as the *Trichoderma reesei* have attracted considerable research interest because of their potential in various industrial applications. However, naturally isolated xylanases cannot withstand harsh conditions such as high temperature and basic pH. In this study, we performed structural analysis of the major *T*. *reesei* xylanase (Xyn2), and novel flexible regions of the enzyme were identified based on B-factor, a molecular dynamics (MD) parameter. To improve thermostability of the Xyn2, disulfide bonds were introduced into the unstable flexible region by using site-directed mutagenesis and two recombinant xylanases, XM1 (Xyn2^Cys12-52^) and XM2 (Xyn2^Cys59-149^) were successfully expressed in *Pichia pastoris*. Secreted recombinant Xyn2 was estimated by SDS-PAGE to be 24 kDa. Interestingly, the half-lives of XM1 and XM2 at 60°C were 2.5- and 1.8- fold higher, respectively than those of native Xyn2. The XM1 also exhibited improved pH stability and maintained more than 60% activity over pH values ranging from 2.0 to 10.0. However, the specific activity and catalytic efficiency of XM1 was decreased as compared to those of XM2 and native Xyn2. Our results will assist not only in elucidating of the interactions between protein structure and function, but also in rational target selection for improving the thermostability of enzymes.

## Introduction

Xylan is a major component of the plant cell wall consisting of a β-D-1,4-linked xylopyranoside backbone substituted with acetyl, arabinosyl, and glucuronosyl side chains [1, 2]. Hydrolysis of the xylan backbone is catalysed by endo-1,4-β-xylanases (EC 3.2.1.8). In recent decades, xylanases have been widely used in many industrial applications and processes, such as in the feed and pulp industries [3, 4]. Moreover, xylanases have also been used in the production of bioethanol [5]. Xylanases are mainly classified into two glycoside hydrolase (GH) families, named family 10 (GH 10) and family 11 (GH 11). Family 10 includes endo-β-1,4-xylanases with higher molecular masses (> 30 kDa) and higher thermostabilities than family 11 xylanases. However, the GH 11 xylanases usually have higher enzymatic activity than the GH 10 xylanases [6]. Previous studies indicated that most xylanases naturally secreted by microorganisms have a moderate optimum temperature range (40–50°C) but cannot withstand temperatures over 50°C (i.e. temperatures used in the pulp and paper industries) [7–9]. Therefore, improving the thermostability of xylanases has attracted considerable research interest worldwide.

Recently, protein engineering techniques such as the random and site-directed mutagenesis were proven to be the most efficient method to improve the thermostability of the GH 11 xylanases [10, 11]. In previous studies, most molecular evolution or protein design was focused on the N-terminus because of its complicated molecular structures [12, 13]. In GH 11 xylanases, protein flexibility plays a critical role in stabilizing the protein structure, and the modification of protein flexibility has been previously utilized to improve the protein stability [14]. In recent years, some flexible molecules or protein regions were successfully identified by using molecular dynamics (MD) simulation. Importantly, these flexible regions were capable of being stabilized by extra intermolecular forces such as salt bridges, hydrogen bonds, aromatic interactions and disulfide bonds [15], which pave the way to improve the protein stability.

*Trichoderma reesei* xylanase 2 (Xyn2) is one of the most important xylanases in the GH 11 family and has a right hand β-sandwich structure [16]. Xyn2 is a low-molecular mass (21 kDa) enzyme with an alkaline isoelectric point (p*I* 9.0) and an optimum activity at pH 4–6. The enzyme rapidly loses its activity when incubated at temperatures over 50°C since it has no stabilizing disulfide bridges or any thermostabilizing domain [16]. In this study, the flexible region at the end of the N-terminal α-helix has been identified based on structural analysis. This flexible region has been stabilized by introducing a covalent disulfide bond (XM1, Cys^14^-Cys^52^). Additionally, the α-helix has been indirectly fixed to the β-core by introducing a disulfide bond between β-sheets B5 and B6 (XM2, Cys^59^-Cys^149^) becuase the α-helix was reported to be associated with the stability of GH 11 family xylanases [17]. We describe the design and expression of the two mutated Xyn2 genes (MX1 and MX2) in *Pichia pastoris*. Moreover, the enzymatic properties of the mutated enzymes are fully characterized.

## Materials and methods

### Microbial strains, culture conditions, and vectors

*Trichoderma reesei* Rut C-30 (ATCC 56765) was cultured in basal medium (0.3% oat spelt xylan, 0.4% KH_2_PO_4_, 1% (NH4)_2_HPO_4_, 1% tryptone, 0.3% yeast extract). *E*. *coli* DH5α was cultured at 37°C in Luria-Bertani medium (1% tryptone, 0.5% yeast extract, and 1% NaCl). *Pichia pastoris* was used as the Xyn2 expression host and was cultured in YPD medium (1% yeast extract, 2% peptone, and 2% glucose). Selection of transformants was performed by using YPD agar containing 100 mg/L zeocin. The pMD19-T Simple vector used for cloning of the Xyn2 gene was purchased from Takara Biotechnology Co., Ltd. (Dalian, China).

### Structural analysis of the *T*. *reesei* Xyn2

The crystal structure with accession code 3AKQ from the RCSB Protein Database (http://www.rcsb.org/pdb/explore/explore.do?structureId=3akq) was identical to Xyn2 in amino acid sequences. The B-factors were calculated by Swiss-PDB viewer (http://spdbv.vital-it.ch/) based on the coordinates from RCSB Protein entry 3AKQ and visualized by using PyMOL 1.7 (http://www.pymol.org/).

### Cloning and site-directed mutagenesis of the Xyn2 gene

The *T*. *reesei* Rut C-30 was cultured in basal medium and subsequently collected for total RNA isolation by TRIzol reagent (Takara Biotechnology Co., Ltd., Dalian) according to the manufacturer’s instructions. First-strand cDNA synthesis was carried out with THE PrimeScript II 1st Strand cDNA Synthesis Kit (Takara Biotechnology Co., Ltd., Dalian) according to the manufacturer’s instructions. Then, the Xyn2 gene was amplified by using the primer pair: P_f_ (5’-GCTGAATTCCAGACGATTCAGCCCGGCA-3’), and P_r_ (5’-ATGCGGCCGCTTAGCTGACGGTGATGGAA-3’), cloned into the pMD19-T Simple Vector and named T-Xyn2. The Fast Mutagenesis System^TM^ (TransGen Biotech Co., Ltd, Beijing) was used for construction of the Xyn2 mutants. All the mutant primers used in this study are listed in Table 1. PCR to introduce mutations was carried by using pfu DNA polymerase (TransGen Biotech Co., Ltd., Beijing) based on the T-Xyn2 template. The desired PCR products were purified by Gel Extraction Mini Kit (Omega). The purified DNA was digested with 1 μl of DMT enzymes (TransGen Biotech Co., Ltd, Beijing) for 3 h and then transformed into *E*. *coli* DH5α. Transformants were selected by using LB agar plates containing 50 μg/mL of ampicillin. A single colony was isolated and inoculated into 3 mL of LB medium containing 50 μg/mL ampicillin, and plasmids were extracted by using the Plasmid Mini Kit (Omega).

**Table 1:** Primers for amino acid mutation.

Enzyme^a^	Mutant site^b^	Primers^c^
XM1	F14C	5’-CGTTCCAGTACGAGTAGCAGTAGCCGTTGTTG-3’ 5’-GCTACTCGTACTGGAACGATGGCCACGGC-3’
	Q52C	5’-TCTTGGTGCCGGGACACCATCCCTTGCCG-3’ 5’-CGGCAAGGGATGGTGTCCCGGCACCAAGA-3’
XM2	V59C	5’-CTGCCCGAGAAGTTGATGCACTTGTTCTTGGTGCCGG-3’ 5’-TGCATCAACTTCTCGGGCAGCTACAACCCCAACGG-3’
	S149C	5’-GTTCGCCGTGTTGACGCAGCCGCTCGAGC-3’ 5’-CGTCAACACGGCGAACCACTTCAACGCGTGGG-3’

^a^ The mutated enzymes include two mutations (XM1: F14C and Q52C; XM2: V59C and S149C)

^b^ Mutation sites are highlighted with underline.

^c^ All the mutation sites in the primers were highlight with underline.

### Expression of the native and mutated Xyn2 in *P*. *pastoris*

The Xyn2 and its mutants, XM1 and XM2, were excised from recombinant T-vectors by double enzyme digestion using *Eco* RI and *Not* I. The target fragments were inserted into the yeast expression vector pPICZαA with the same sticky ends, followed by transformation into *E*. *coli* DH5α. The recombinant shuttle plasmid pPICZαA was linearized with *Sal* I and electroporated into *P*. *pastoris*. All *P*. *pastoris* transformants were cultured and induced in buffered minimal methanol medium (BMMY) at 30°C for 72 h. The supernatant of each xylanase was dialysed against McIlvaine buffer (0.2 M sodium phosphate, 0.1 M citric acid, pH 6.0). The dialysate was concentrated to 1 mL by ultrafiltration using a 10 kDa cut-off membrane (Sangon Biotech, Shanghai).

### SDS-PAGE analysis

The protein fraction (cell culture supernatant) was boiled for 5 min and then applied to a 12% (w/v) SDS-PAGE gel. The proteins were visualized using Coomassie Brilliant Blue R 250 staining. The total protein concentration was determined by the Bradford method using bovine serum albumin (BSA) as a standard.

### Enzymatic activity assays

The activity of the endo-1,4-β-xylanases was determined by using the 3, 5-dinitrosalicylic acid (DNS) colorimetric method. In brief, 40 μl of enzyme diluted with McIlvaine buffer (0.2 M sodium phosphate, 0.1 M citric acid, pH 6.0) was added to 360 μl of 1% (w/v) beech wood xylan (Sigma) that was suspended with the same buffer and incubated at 50°C for 10 min. The reducing sugars hydrolysed by xylanase were quantitated by adding 600 μl of DNS and boiling at 100°C for 10 min. The absorbance of the solution was then measured at 540 nm by using a visible-spectrophotometer. One unit (IU) of xylanase activity was defined as the amount of enzyme that liberated 1 μmol of reducing sugar equivalent per minute under standard conditions (at pH 6.0 and 50°C for 10 min).

### Validation of disulfide bond formation

The formation of disulfide bonds of the recombinant proteins was measured by using the method described by Yang [18]. In brief, the native and mutated enzymes proteins were pre-treated with a concentration gradient of 1,4-Dithiothreitol (DTT) ranging from 1–5 mM in the presence of 1% SDS at 70°C for 5 min, and then applied to a 12% (w/v) SDS-PAGE gel. To determine the influence of disulfide bond introduction on enzyme thermostability, the purified enzymes were pre-treated with 10 mM DDT at 4°C for 12 h, and then the residue activity of the enzymes was measured after incubation at 60°C for 10 min.

### Glycosylation prediction and validation of recombinant proteins

The glycosylation of Xyn2 and its mutants was predicted by the ExPASy SIB Bioinformatics Resource Portal (http://www.expasy.org/) with the PROSITE program (http://prosite.expasy.org/) based on the amino acid sequences. Moreover, glycosylation of the recombinant proteins was experimentally verified by SDS-PAGE using purified proteins with or without being pre-treated with endo-glycosidase (Endo H_f_).

### Enzymatic property assays

The optimal temperature was measured by performing the xylanase activity assay at temperatures ranging from 30 to 80°C. The thermostability of the xylanases was measured by pre-incubating the xylanases in 50 mM McIlvaine buffer (pH 6.0) for 10 min at different temperatures (50 to 80°C), and residue enzyme activity was measured after cooling on ice. Enzymatic assays at different pH values were performed at the optimal temperature over a pH range of 2.0–10.0. The buffers used were 50 mM McIlvaine buffer (pH 2.0–8.0) and 50 mM of a glycine–NaOH buffer (pH 9.0–10.0). To determine the half-lives (t_1/2_) of the xylanases, the proteins were incubated in McIlvaine buffer (pH 6.0) at 60°C, and the residue activity was measured at different time intervals (1, 3, 5, 10, 15, 20, and 30 min). The half-life was calculated by using the equation (1. *y* = A*e^*-kt*^), which was fitted to specific activity-time curves with Excel and the equation t_1/2_ = ln2/k. The *K*m and *k*_cat_ values were determined by using the method as described by Jiang et al [19].

## Results and discussion

### Identification of the flexible regions in the *T*. *reesei* Xyn2

**Fig 1:**
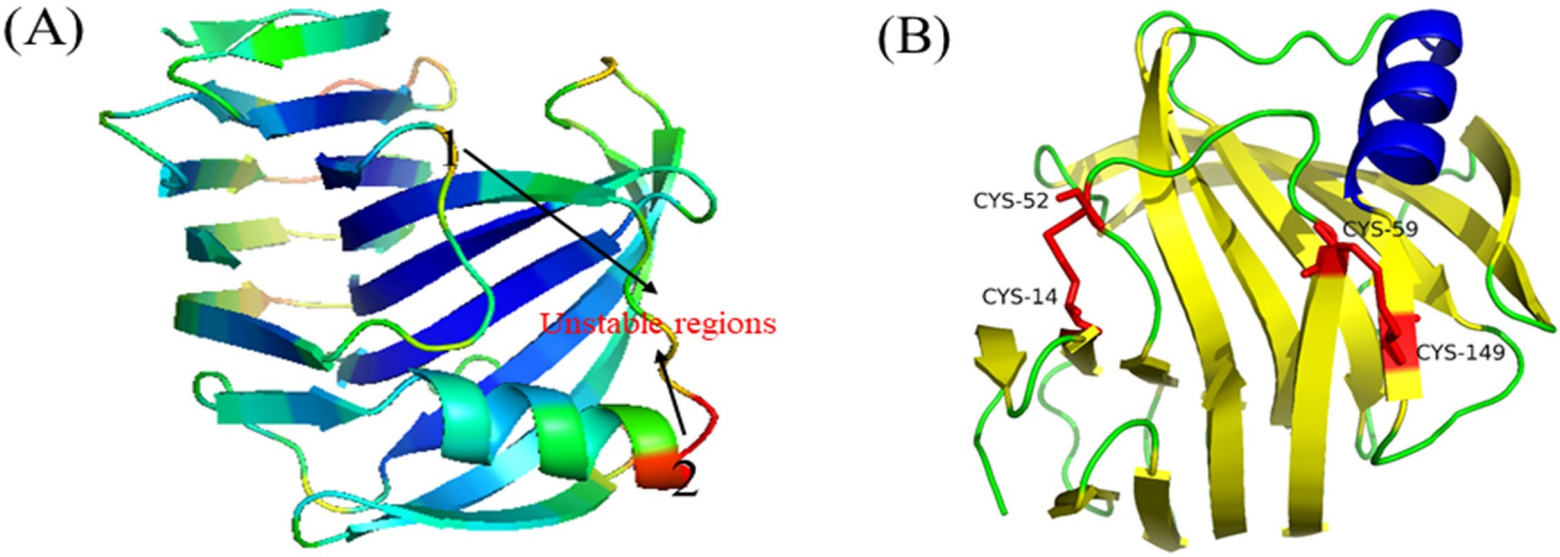
Structural analysis of *T*. *reesei* Xyn2 and molecular design. (A)Visualization of unstable regions of Xyn2; (B) Locations of the predicted disulfide bond in Xyn2.

Flexible regions have been recognized as critical unstable factors for proteins, and the modulation of flexible regions has been previously used to improve the protein stability [15]. However, more studies have focused on engineering the protein termini [12, 20], since it is difficult to obtain sufficient information about protein molecular structures. Therefore, the N-terminus was usually selected as the target for protein engineering to improve the thermostability of GH 11 xylanases [12, 21]. In addition to the N-terminus, other critical domains or regions related to the stability of the GH 11 xylanases are still unknown. Currently, molecular dynamics simulation offers an efficient approach to identify the flexible regions of proteins [22, 23]. In this work, the flexible regions of Xyn2 were identified based on the B-factor, molecular dynamics parameter. The B-factor of the amino acids in Xyn2 were visualized by PyMOL with colors ranging from blue to red (Fig 1A). We show that the fragments of a β-sheet (A3/B5) and an α-helix exhibited larger B-factors than those from other regions of Xyn2. The two regions were selected for protein engineering.

### Amino acid mutations in the flexible regions of Xyn2

Previous studies indicated that flexible regions in the protein could be stabilized by the intermolecular forces such as the salt bridges, hydrogen bonds, and aromatic interactions [15]. However, covalent disulfide bonds have been considered to be the most important structures responsible for protein stability [24]. In this study, disulfide bonds were engineered into the flexible regions of Xyn2 by site-directed mutagenesis (Fig 1B). A 600 bp DNA fragment was successfully amplified by using a pair of specific primers. The DNA sequences were subsequently aligned by NCBI, and the results indicated that the desired DNA were identical to *T*. *reesei* Rut C-30 Xyn2 (Gen Bank Accession No.EU532196.1). The mutated Xyn2 genes (XM1 and XM2) were constructed by using Fast Mutagenesis System. The mutated genes were sequenced, and the results showed that both genes have a sequence similar to that of the Xyn2 gene (S1 Fig). As expected, the mutated amino acids (XM1, Cys^14^-Cys^52^; XM2, Cys^59^-Cys^149^) were successfully introduced into the *T*. *reesei* Xyn2. Both genes encode a mature, 190-amino acid Xyn2 protein (S2 Fig). The calculated molecular weight (21 kDa) is consistent with the molecular weight of native Xyn2 isolated from *T*. *reesei* [16].

### Expression of the Xyn2 and its mutants in *P*. *pastoris*

**Fig 2:**
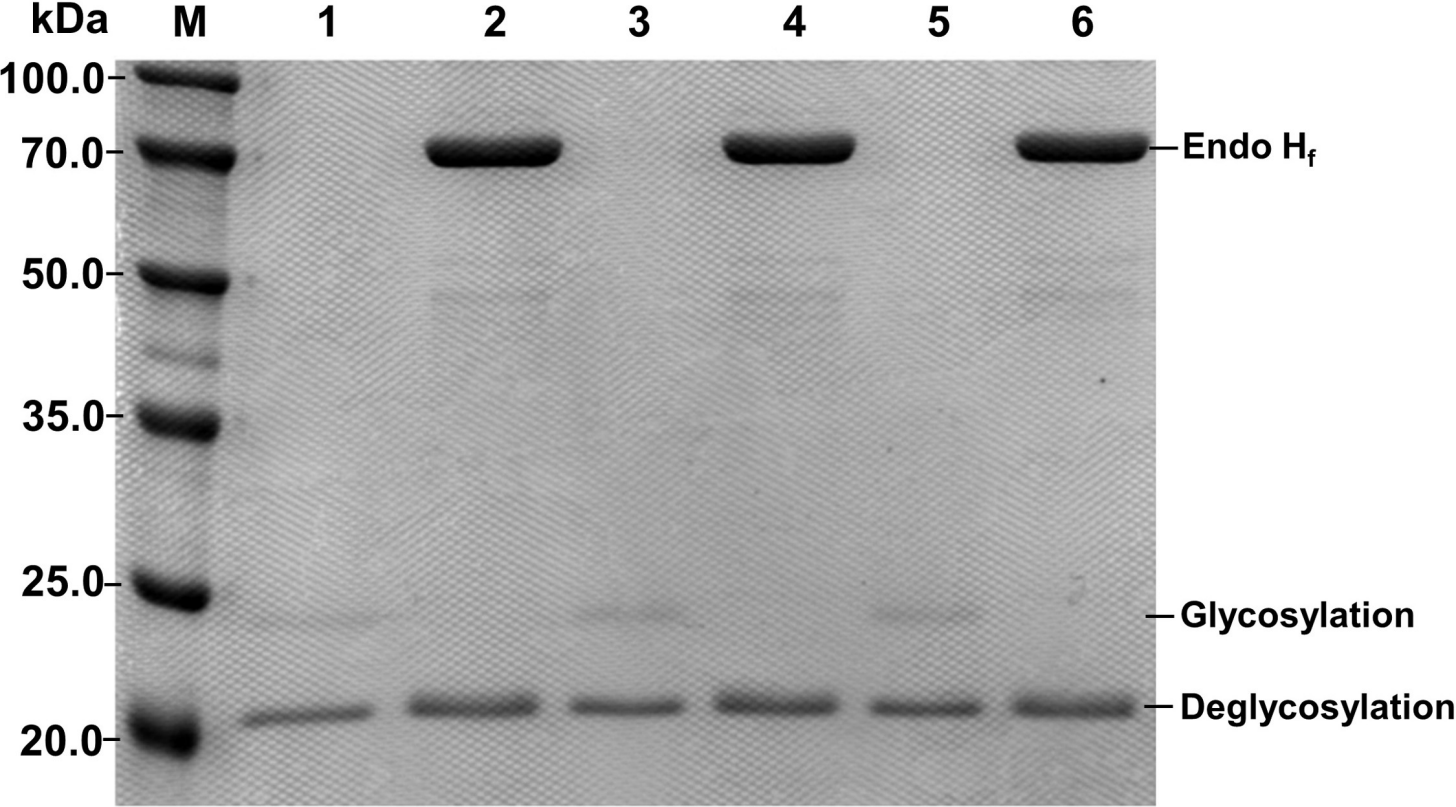
SDS-PAGE analysis of Xyn2 and its mutants. M: Mark; Lanes 1, 3, 5: Expression product of xyn2, XM1 and XM2; Lanes 2, 4, 6: Expression product of xyn2, XM1 and XM2 treated with Endo H_f_.

**Fig 3:**
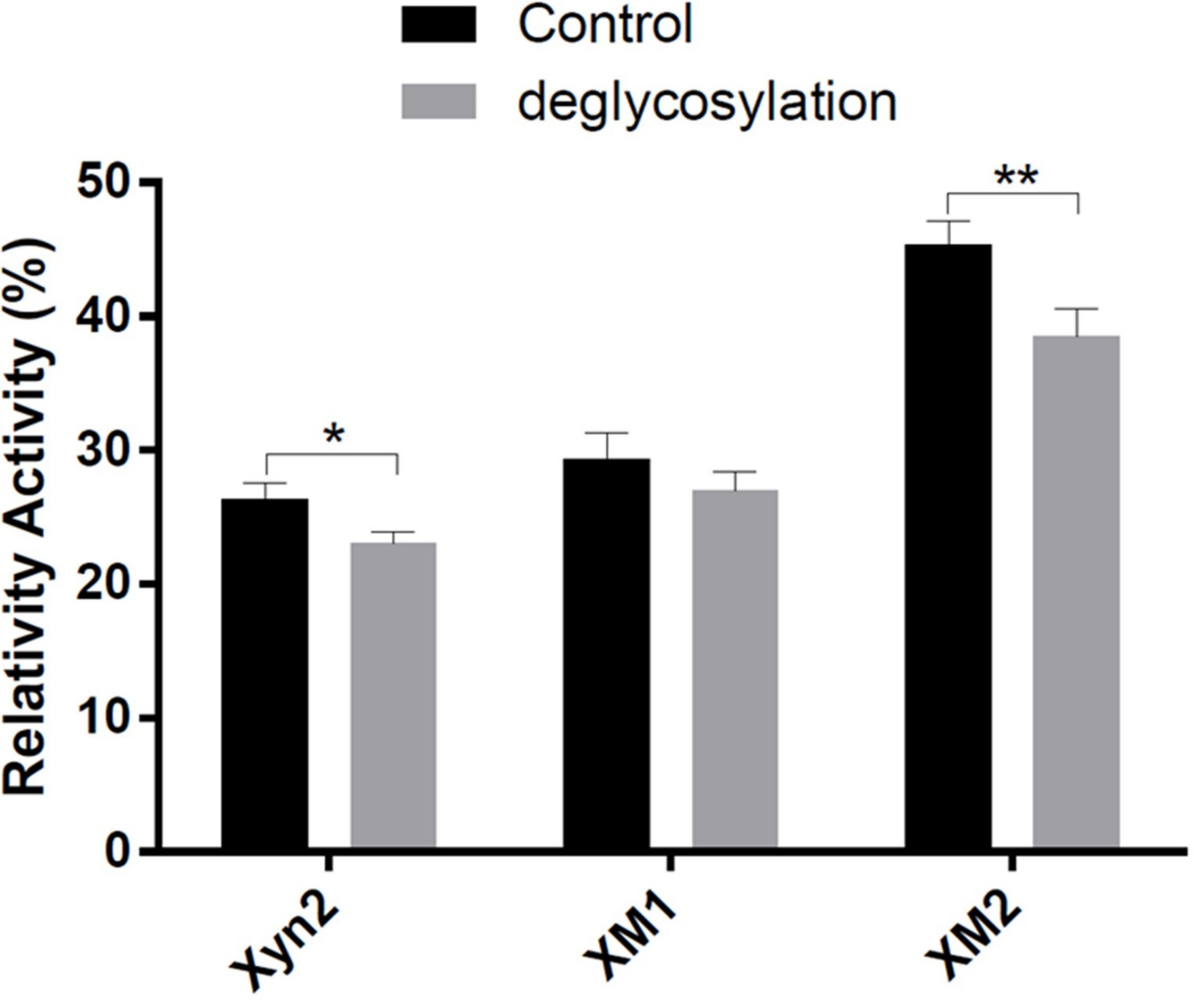
Influence of glycosylation on the enzymatic activity of Xyn2 and mutated enzymes. Control: untreated enzymes; Deglycosylation: enzymes treated with endo H_f_.

The *P*. *pastoris* is the most important heterologous expression host strain used in industrial or medical fields because of several advantages such as promoter strength and ease of achieving a high cell density [25, 26]. In this study, the native Xyn2 gene and its mutants (XM1 and XM2) were integrated into the genome of *P*. *pastoris* X-33 by the pPICZαA shuttle plasmid and successfully expressed in *P*. *pastoris* X-33 (Fig 2). A previous study indicated that the molecular weight of the native *T*. *reesei* Xyn2 was 21 kDa. However, the Xyn2 enzyme secreted by *P*. *pastoris* has a different molecular weight molecular. The molecular weight of Xyn2 secreted by *P*. *pastoris* is 24 kDa as estimated by SDS-PAGE. There is a 3 kDa difference in the molecular weights of Xyn2 proteins secreted by *P*. *pastoris* and *T*. *reesei*. This difference is caused by N-glycosylation of the proteins since the *P*. *pastoris* tends to hyperglycosylate heterologous proteins [27–30]. Treatment of the recombinant Xyn2 protein with endoglycosidase F produced a novel protein species with a molecular weight of 21 kDa (Fig 2), which is consistent with the native Xyn2 secreted by *T*. *reesei*. However, this large, glycosylated protein was efficiently secreted and passed through the yeast cell wall into the culture medium. We also performed the glycosylation site analysis by using the PROSITE program (http://prosite.expasy.org/). We found that the mutated amino acids were not involved in the glycosylation sites, suggesting that glycosylation did not affect the formation of disulfide bonds in mutated Xyn2. However, the glycosylation seemed to affect the activity of the enzymes, and the enzymatic activity of the recombinant proteins was decreased after deglycosylation (Fig 3). This finding is consistent with previous studies showing that glycosylation is required for the full activity of a wide variety of enzymes [31, 32]. The influence of glycosylation on protein properties may be attributed to several functions such as ensuring correct protein folding, preventing proteolytic degradation, and facilitating intracellular transportation [31]. Moreover, glycosylation is one of the greatest advantages of the yeast expression systems [25, 26].

**Fig 4:**
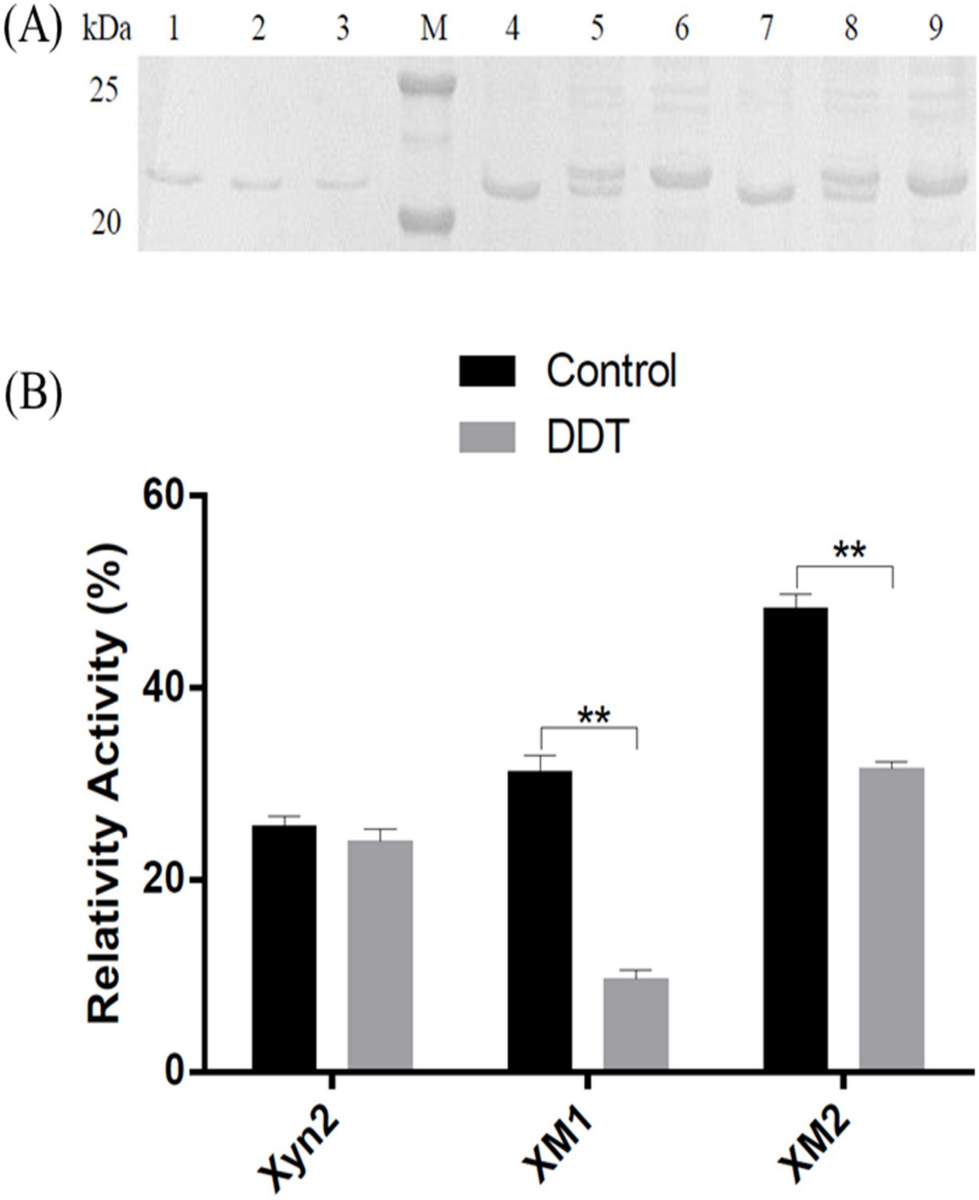
Validation of disulfide bonds in XM1 and XM2. (A) SDS-PAGE. M: Marker; Lane 1, 2, and 3: the native Xyn2 were treated with 0, 2.5, and 10 mmol DDT; Lane 4: XM1; Lane 5: XM1 treated with 2.5 nmol DDT; Lane 6: XM1 treated with 10 nmol DDT; Lane 7: XM2; Lane 8: XM2 treated with 2.5 mmol DDT; Lane 9: XM2 treated with 10 mmol DDT. (B) Influence of amino acid mutation on enzymatic activity.

To validate the formation of the engineered disulfide bonds, the purified enzymes were pre-treated with DDT before SDS-PAGE. The presence of disulfide bonds changes the mobility of proteins, and proteins without disulfide bonds tended to bind more SDS, resulting in a decreased migration speed compared to that of proteins with intact disulfide bonds [18]. As shown in Fig 4A, both the XM1 and XM2 moved slowly on the gels after DDT treatment, indicating that disulfide bonds were successfully introduced into XM1 and XM2. Interestingly, the introduction of disulfide bonds affected the activity of Xyn2 and abolishing the disulfide bonds by using DDT resulted in a significant reduction in its activity (Fig 4B). This finding is consistent with a previous report that disulfide bonds can serve as a switch for protein functions and introducing disulfide bonds to a functional protein may increase both its stability and activity [33].

### Effect of amino acid mutation on thermostability and pH stability

**Fig 5:**
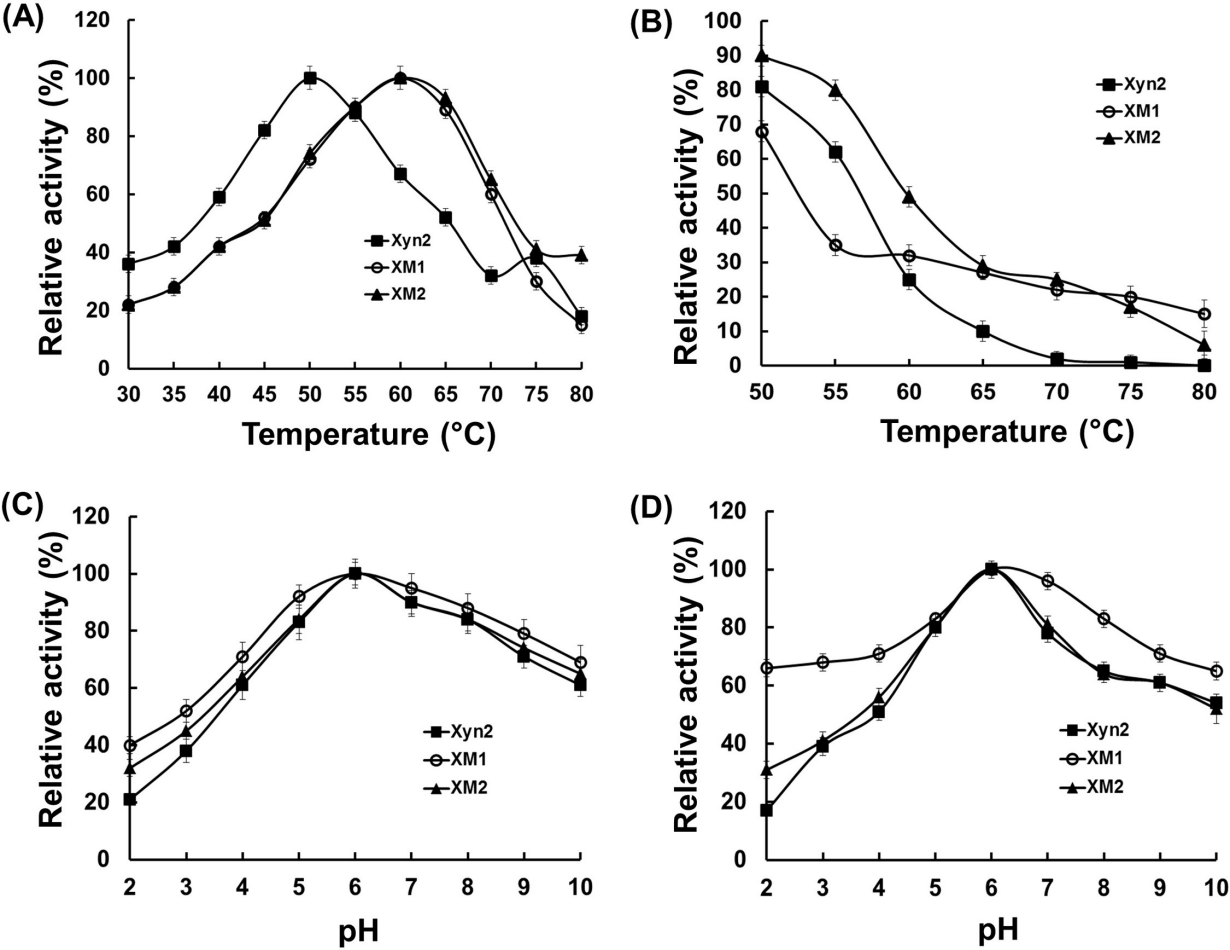
Enzymatic properties of the Xyn2 and mutated enzymes. (A) Influence of temperature on the enzyme activity; (B) The thermostability of the enzymes; (C) Influence of pH on the enzyme activity; (D) The pH stability of the enzymes.

Currently, there are two approaches to obtain thermostable xylanases. The first is to discover enzymes from thermophilic microorganisms, and the second is to engineer currently used mesophilic xylanases to create novel enzymes that can withstand the harsh conditions [4]. However, the isolation of thermostable xylanases is a very tedious and difficult task because of their low expression levels. Previous studies indicated that the covalent disulfide bonds played a critical role in stabilizing the flexible parts of proteins, and introducing extra disulfide bonds has successfully improved the thermostability of many protein species [17, 34]. However, most studies engineered disulfide bonds on the protein surface or at the N and C terminus. In the present study, we identified an unstable flexible region of Xyn2, and disulfide bonds were introduced to stabilize this region. Compared to native Xyn2, the optimal temperature for the two mutants changed from 50°C to 55°C (Fig 5A) and both proteins retained more activity after incubation at this temperature than native Xyn2. Interestingly, native Xyn2 lost nearly all activity after incubation at 70°C for 10 min. However, both mutated proteins retained approximately 30% activity (Fig 5B). Moreover, the half-lives of XM1 and XM2 at 60°C increased 2.5- and 1.8-fold, respectively (Table 2). Both of their half-lives at this temperature were higher than those from previous reported enzymes engineered by Wakarchuk et al [17].

The N-terminus of the GH 11 family xylanases is recognized as a thermostable domain because of its crucial role in stabilizing the β-core, and unfolding of the N-terminus caused direct exposure of the β-core to thermal or acidic conditions [12, 13]. Moreover, an α-helix also plays a critical role in stabilizing the GH 11 family xylanases, and the introduction of disulfide bonds to this α-helix significantly increased the stability of xylanases from *Bacillus circulans* [17]. In this study, the identified unstable region involves both the β-core and the α-helix, and the introduction of disulfide bonds stabilized this region, which led to improvements in the thermostability of *T*. *reesei* Xyn2. Interestingly, the introduction of disulfide bonds did not affect the optimal pH of Xyn2 (Fig 5C). However, the XM1 showed a broader pH range than native Xyn2 and XM2, and it retained more than 60% activity at a pH range of 2.0 to 10.0 (Fig 5D). The result is consistent with a previous report in which introducing disulfide bonds decreased pH sensitivity by reducing the electronic density on the surface of the protein [18]. The thermostability of XM1 and XM2 was lower than several xylanases naturally secreted by thermophilic microorganisms such as *Thermotoga maritima* and *Thermomyces lanuginosus* [19, 35]. However, the *P*. *pastoris* heterologous expression system allows higher expression levels than expression in natural microorganisms [36].

**Table 2:** Comparison of enzymatic properties of Xyn2, XM1 and XM2.

Enzymatic properties^a^	Xyn2	XM1	XM2
Specific activity (U/mg)	1037.92	470.73	934.17
pH stability^b^	5–9	2–10	5–9
t_1/2_ at 60°C (min)	4.4	10.7	7.5
*K*_m_ (mg/ml)	4.5	15.4	6.9
*kcat* (S^-1^)	89.4	179.2	97.2

^a^ Enzymes were diluted to the similar concentration for enzymatic properties analysis and measured in triplicate.

^b^Retained more than 60% maximal activity.

### Effect of amino acid mutations on enzymatic activity and kinetic parameters

Changes in the amino acids or conformation of an enzyme protein may result in altered properties such as enzymatic activity and kinetic parameters [37]. The catalytic domains of GH 11 family xylanases consist principally of β-pleated sheets formed into a two-layered trough that surrounds the catalytic site [38, 39]. Additionally, the N-terminus of *T*. *reesei* xylanases is known to be associated with their thermostability and catalytic activity [27]. In the present study, disulfide bond introduction at the N-terminus (Cys^14^-Cys^52^) decreased the specific activity of the enzyme (Table 1). Both the *K*_m_ (15.4 mg/ml) and *k*_cat_ (179.2 s^-1^) of XM1 were significantly higher than those of native Xyn2. In contrast, fixing the α-helix to the β-core through a disulfide bond between β-sheets B5 and B6 (Cys^59^-Cys^149^) had little influence on the catalytic efficiency of Xyn2. The decreased catalytic efficiency of XM1 may be associated with the substrate binding capacity since previous studies indicated that the N-terminus of GH 11 family xylanases plays a critical role in substrate binding because of the presence of charged amino acid residues [14, 40]. The electronic charges on the surface of the N-terminus may be covered by the introduced disulfide bonds.

## Conclusions

In this study, we successfully introduced disulfide bonds into the unstable flexible regions of *T*. *reesei* Xyn2 by using site-directed mutagenesis, and two mutated xylanases were successfully expressed in *Pichia pastoris*. Introduction of the disulfide bonds resulted in elevated thermostability and pH stability in the *T*. *reesei* Xyn2. However, disulfide bonds introducing at the N-terminal may affect the catalytic efficiency. Our results not only identified a novel unstable region in the *T*. *reesei* xylanases but also offer a potential avenue to improve the stability of the enzyme.

## Supporting information

S1 Fig Comparison of the nucleotide sequences of the native Xyn2 and the mutated Xyn2 (the mutation has been highlighted in red). (TIFF)

S2 Fig Comparison of the deduced amino acid sequences of the native Xyn2 and mutated Xyn2. (TIFF)

## References

[1] Puls J, Schusell J (1993) Chemistry of hemicelluloses: relationship between hemicellulose structure and enzymes required for hydryolsis, p.1–27. In Coughlan MP and Hazlewood GP (ed.), Hemicellulose and hemicelluloases. Portland Press, London.

[2] Beg QK, Kapoor M, Mahajan L, Hoondal GS (2001) Microbial xylanases and their industrial applications: a review. Appl Microbiol Biotechnol 56:326–338. 11548999 10.1007/s002530100704

[3] Kumar V, Dangi AK, Shukla P (2018) Engineering thermostable microbial xylanases toward its industrial applications. Mol Biotechnol 60:226–235. 10.1007/s12033-018-0059-6 29380253

[4] Jun H, Jia Y, Li W, Bing Y, Daiwen C (2010) Functional characterization of a recombinant xylanase from *Pichia pastoris* and effect of the enzyme on nutrient digestibility in weaned pigs. Brit J Nutr 203: 1507–1513. 10.1017/S0007114509993333 20064285

[5] Taherzadeh MJ, Karimi K (2008) Pretreatment of Lignocellulosic Wastes to Improve Ethanol and Biogas Production: A Review. Int J Mol Sci 9:1621–1651. 10.3390/ijms9091621 19325822 PMC2635757

[6] Sriprang R, Asano K, Gobsuk J, Tanapongpipat S, Champreda V, Eurwilaicitr L. (2006) Improvement of thermostability of fungal xylanase by using site-directed mutagenesis. J Biotechnol 126:454–462. 10.1016/j.jbiotec.2006.04.031 16757052

[7] Basu M, Kumar V, Shukla P (2018) Recombinant approaches for microbial xylanases: recent advances and perspectives. Curr Potein Pept Sci 19:87–99. 10.2174/1389203718666161122110200 27875966

[8] Biely P, Vrsanská M, Tenkanen M, Kluepfel D (1997) Endo-beta-1,4-xylanase families: differences in catalytic properties. J Biotechnol 57:151–166. 9335171 10.1016/s0168-1656(97)00096-5

[9] Paes G, Berrin JG, Beaugrand J (2012) GH11 xylanases: Structure/function/properties relationships and applications. Biotechnol adv, 30:564–592. 10.1016/j.biotechadv.2011.10.003 22067746

[10] Satyanarayana DV (2013) Improvement in thermostability of metagenomic GH11 endoxylanase (Mxyl) by site-directed mutagenesis and its applicability in paper pulp bleaching process. J Ind Microbiol Biotechnol 40:1373–1381. 10.1007/s10295-013-1347-6 24100791

[11] Baweja M, Nain L, Kawarabayasi Y, Shukla P (2016) Current technological improvements in enzymes toward their biotechnological applications. Front Microbiol 7: 965 10.3389/fmicb.2016.00965 27379087 PMC4909775

[12] Zhang S, Zhang K, Chen X, Chu X, Sun F, Dong ZY (2010) Five mutations in N-terminus confer thermostability on mesophilic xylanase. Biochem Bioph Res Co 395:200–206. 10.1016/j.bbrc.2010.03.159 20361933

[13] Hao-Meng Y, Kun M, Hui-Ying L, Ya-Ru W, Tie-Zheng Y, Guo-Ying B, et al (2006) Improvement of the thermostability of xylanase by N-terminus replacement. Chinese J Biotechnol 22:26–32. 16572836

[14] Joo JC, Pack SP, Kim YH, Yoo YJ (2011) Thermostabilization of Bacillus circulans xylanase: computational optimization of unstable residues based on thermal fluctuation analysis. J Biotechnol, 151:56–65. 10.1016/j.jbiotec.2010.10.002 20959126

[15] Kumar S, Tsai CJ, Nussinov R. (2000) Factors enhancing protein thermostability. Protein Eng 13:179 10775659 10.1093/protein/13.3.179

[16] Turunen O, Etuaho K, Fenel F, Vehmaanperä J, Wu X, Rouvinen J, et al (2001) A combination of weakly stabilizing mutations with a disulfide bridge in the α-helix region of Trichoderma reesei endo-1,4-β-xylanase II increases the thermal stability through synergism. J Biotechnol 88:37 11377763 10.1016/s0168-1656(01)00253-x

[17] Wakarchuk WW, Sung WL, Campbell RL, Cunningham A, Watson DC, et al (1994) Thermostabilization of the Bacillus circulansxylanase by the introduction of disulfide bonds. Protein Eng 7:1379–1386. 7700870 10.1093/protein/7.11.1379

[18] Yang HM, Yao B, Meng K, Wang YR, Bai YG, Wu NF (2007) Introduction of a disulfide bridge enhances the thermostability of a Streptomyces olivaceoviridis xylanase mutant. J Ind Microbiol Biotechnol 34:213–218. 10.1007/s10295-006-0188-y 17139507

[19] Bhardwaj A, Leelavathi S, Mazumdar-Leighton S, Ghosh A, Ramakumar S, Reddy VS (2010) The critical role of N-and C-terminal contact in protein stability and folding of a family 10 xylanase under extreme conditions. PloS one 5:e11347 10.1371/journal.pone.0011347 20596542 PMC2893209

[20] Wang Y, Fu Z, Huang H, Zhang H, Yao B, Xiong HR, et al (2012) Improved thermal performance of Thermomyces lanuginosus GH11 xylanase by engineering of an N-terminal disulfide bridge. Bioresour Technol 112:275–279. 10.1016/j.biortech.2012.02.092 22425398

[21] Zhang H, Li J, Wang J, Yang Y, Wu M (2014) Determinants for the improved thermostability of a mesophilic family 11 xylanase predicted by computational methods. Biotechnol Biofuels 7:1 10.1186/1754-6834-7-1 24393334 PMC3895927

[22] Somayesadat B, Bevan DR, Zhang C (2012) Study and design of stability in GH5 cellulases. Biotechnol Bioeng 109:31–44. 10.1002/bit.23280 21809329

[23] Creighton TE (1992) What the papers say: Protein folding pathways determined using disulphide bonds. Bioessays 14:195–199. 10.1002/bies.950140310 1285385

[24] Buckholz RG, Gleeson MAG (1991) Yeast Systems for the Commercial Production of Heterologous Proteins. Biotechnol 9:1067. 10.1038/nbt1191-1067 1367623

[25] Romanos MA, Scorer CA, Clare JJ (1992) Foreign gene expression in yeast: a review. Yeast 8:423–488. 10.1002/yea.320080602 1502852

[26] Törrönen A, Mach RL, Messner R, Gonzalez R, Kalkkinen N, Harkki A, et al (1992) The two major xylanases from *Trichoderma reesei*: characterization of both enzymes and genes. Nat Biotechnol 10:1461–1465. 10.1038/nbt1192-1461 1369024

[27] Hamilton SR, Gerngross TU (2007) Glycosylation engineering in yeast: the advent of fully humanized yeast. Curr Opin Biotech 18:387–392. 10.1016/j.copbio.2007.09.001 17951046

[28] Hamilton SR, Zha D (2015) Progress in Yeast Glycosylation Engineering. Methods Mol Biol 1321:73–90. 10.1007/978-1-4939-2760-9_6 26082216

[29] Jiang Z, Zhu Y, Li L, Yu X, Kusakabe I, et al (2004) Transglycosylation reaction of xylanase B from the hyperthermophilic Thermotoga maritima with the ability of synthesis of tertiary alkyl beta-D-xylobiosides and xylosides. J Biotechnol 114:125–134. 10.1016/j.jbiotec.2004.05.007 15464606

[30] Seelhorst K, Stacke C, Ziegelmuller P, Hahn U (2013) N-Glycosylations of human α1, 3-fucosyltransferease IX are required for full enzyme activity. Glycobiology 23: 559–567. 10.1093/glycob/cws219 23263199

[31] Muta K, Fukami T, Nakajima M, Yokoi T (2014) N-Glycosylation during translation is essential for human arylacetamide deacetylase enzyme activity. Biochem Pharmacol 87: 352–359. 10.1016/j.bcp.2013.10.001 24125761

[32] Hogg PJ (2003) Disulfide bonds as switches for protein function. Trends Biochem Sci 28: 210–214. 10.1016/S0968-0004(03)00057-4 12713905

[33] Matsumura M, Matthews BW (1989) Control of Enzyme Activity by an Engineered Disulfide Bond. Science 243:792–794. 2916125 10.1126/science.2916125

[34] Jiang ZQ, Deng W, Zhu YP, Li LT, Sheng YJ, Hayashi K (2004) The recombinant xylanase B of *Thermotoga maritima* is highly xylan specific and produces exclusively xylobiose from xylans, a unique character for industrial applications. J Mol Catalysis B: Enzymatic 27:207–213.

[35] Kumar V, Chhabra D, Shukla P (2017) Xylanase production from *Thermomyces lanuginosus* VAPS-24 using low cost agro-industrial residues via hybrid optimization tools and its potential use for saccharification. Bioresource Technol 243, 1009–1019 10.1016/j.biortech.2017.07.094 28764103

[36] Buckholz RG, Gleeson MAG (1991) Yeast systems for the commercial production of heterologous protein. Biotechnol 9:1067–1072. 10.1038/nbt1191-1067 1367623

[37] Roberge M, Shareck F, Morosoli R, Kluepfel D, Dupont C (1999) Characterization of active-site aromatic residues in xylanase A from Streptomyces lividans. Protein Eng 12:251–257. 10235626 10.1093/protein/12.3.251

[38] Miao SC, Ziser L, Aeersold R, Withers SG (1994) Identification of glutamic acid 78 as the active site nucleophile in Bacillus subtilis xylanase using electrospray tandem mass spectrometry. Biochemistry 33: 7027–7032. 7911679 10.1021/bi00189a002

[39] Withers SG, Aebersold R (1995) Approaches to labeling and identification of active-site residues of glycosidases. Protein Sci 4: 361–372. 10.1002/pro.5560040302 7795519 PMC2143074

[40] Ludwiczek ML, Heller M, Kantner T, McIntosh LP (2007) A secondary xylan-binding site enhances the catalytic activity of a single-domain family 11 glycoside hydrolase. J Mol Biol 373:337–354. 10.1016/j.jmb.2007.07.057 17822716

