# Supplementary figures and images for: Design, expression and functional characterization of a thermostable xylanase from Trichoderma reesei
Source: PLoS One. 2019 Jan 16;14(1):e0210548. doi: 10.1371/journal.pone.0210548 (PMC6334952; doi:10.1371/journal.pone.0210548)

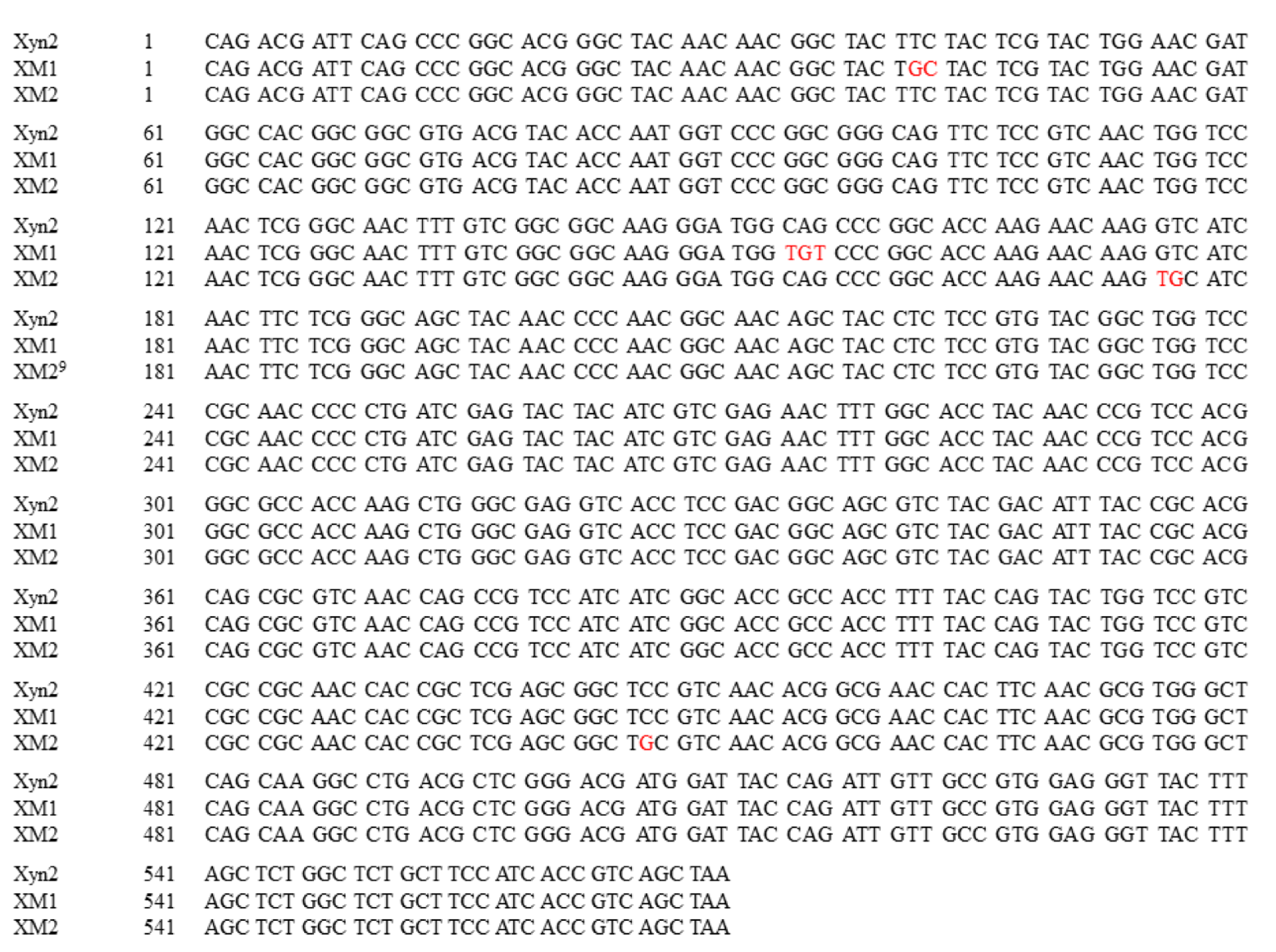

Supplement: S1 Fig — (TIFF) [file pone.0210548.s001.tiff]

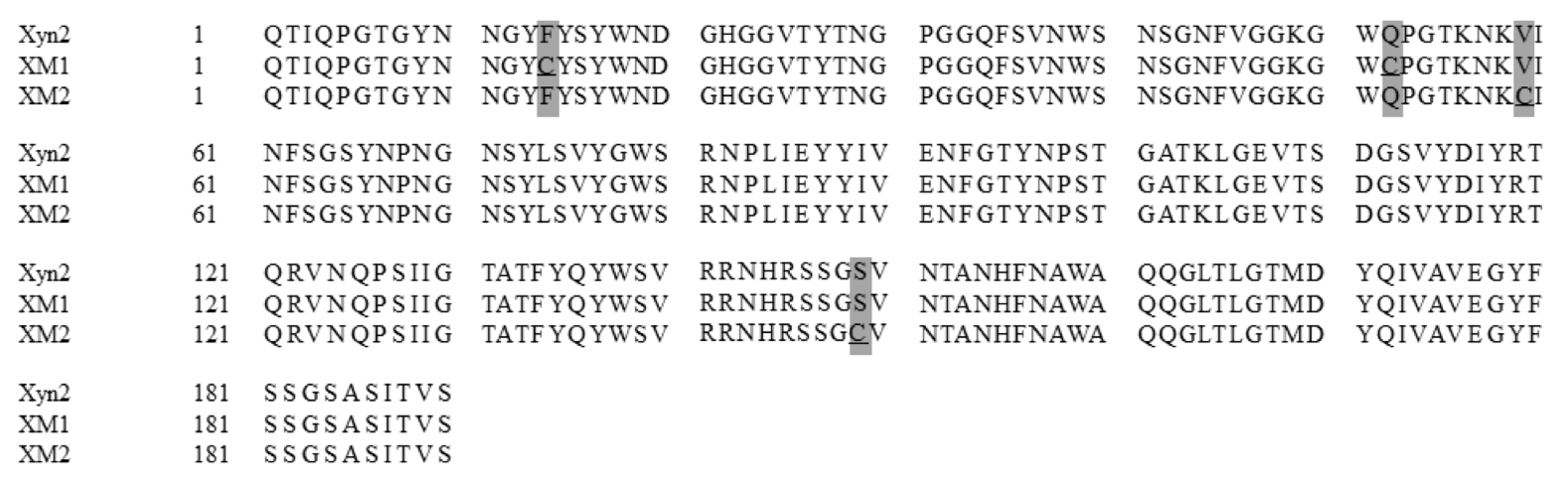

Supplement: S2 Fig — (TIFF) [file pone.0210548.s002.tiff]
